# Editorial: Industry and individuals: branding, labelling, and marketing of food products

**DOI:** 10.3389/fnut.2025.1555875

**Published:** 2025-01-28

**Authors:** Daniel Adrian Gârdan, Paweł Bryła, Ionel Dumitru, Iuliana Petronela Gârdan

**Affiliations:** ^1^Department of Economic Sciences, Faculty of Economic Sciences, Spiru Haret University, Bucharest, Romania; ^2^Doctoral School of Economic Sciences, “Dunărea de Jos” University of Galaţi, Galaţi, Romania; ^3^Department of International Marketing and Retailing, Faculty of International and Political Studies, University of Lodz, Lodz, Poland; ^4^Department of Marketing, Faculty of Marketing, Bucharest University of Economic Studies, Bucharest, Romania; ^5^Department of Psychology and Education Sciences, Faculty of Psychology and Education Sciences, Spiru Haret University, Bucharest, Romania

**Keywords:** food branding, food labeling, food packaging, dietary choices, consumer behavior

Some of the most important changes nowadays are those that concern our eating behavior. In this decision-making process, a whole series of factors shape our perceptions, attitudes and behavior. These factors are in turn the expression of radical transformations based on the advance of technologies, changes caused by the post-pandemic crisis, environmental challenges and substantive changes in the global economy. Our Research Topic proposed to take a look from various perspectives and various scientific backgrounds into these factors and the way in which consumers are responding to certain stimulus. Studies have shown that consumers are sensitive to sustainable labeling and trusted sources such as governments or public authorities that can back up the labeling process (1). Labeling that uses different simple and intuitive cues is more efficient and elicits a faster response in terms of consumers' decisions. We observe efforts made in the legislative field to help food consumers to make more informed and healthier choices based on the food products front labeling (2, 3). A sustainability labeling framework (that can include regulatory measures for green claims, managing efficiently origin labeling, etc.) is needed and seen as an essential solution for the optimization of food consumers perceptions (2, 4). Consumers are often sensitive to information and stimuli that can raise their level of self-awareness and positive self-image. Food and packaging-related eco-labels have been highlighted as extrinsic cues able to affect consumers' perception of food quality and safety (5). There is a need to investigate reading labels in fine-grained models, adapted to different types of labels and different contexts of reading (6). The level of consumers' trust, and by extension, their positive decisions toward healthier food products, can be optimized through a substantial effort to inform them about the new smart packaging technology and its benefits (7). This effort is educational in its nature—consumers being taught how to recognize proper nutritional information and how to combine it in order to maximize their consumption (along with budgetary concerns and other consumption barriers that can prevail in certain situations).

In our complex global environment, dietary choices are more and more influenced by factors like nutritional information, brand values, package design etc. The papers from the “*Industry and Individuals: Branding, Labelling, and Marketing of Food Products*” Research Topic are exploring these factors from a diversified perspective. The aim of the Research Topic was to highlight the complexity of these effects on food consumers, unraveling the interplay between industries and consumer behaviors, their food choices, and purchasing decisions. The papers within the Research Topic proposed new insights from various scientific backgrounds. Seyedhamzeh et al. explore the degree of effectiveness associated with new labeling strategies that are pointing toward the promotion of healthier dietary choices. The paper is referring to an important theme that can be found also in subsequent papers—the importance of transparent and innovative labeling from the point of view of the effect upon consumer behavior. In the paper by Mulligan et al., the focus shifts to a very interesting topic—the influence of cartoon characters used on food packaging upon the children's food choices. This article complements the first by highlighting the specific impact of marketing strategies on young consumers, in the field of food products, highlighting also the need for regulation and ethical marketing practices, when it comes to products addressed to children. Paştiu et al. expand the discussion toward the problems of accessibility in terms of food choices exploring how economic and availability factors in rural settings may influence or affect dietary decisions. Results of their research emphasize once more the importance of informative labeling and ethical marketing in ensuring fair access to nutritional information.

Kikuta et al. further investigates the complex process of building trust in food products through the use and promotion of proper accredited food labels. Authors are emphasizing the role of governmental and organizational oversight to maintain these standards that are important factors capable of influencing consumer decision-making. Trustworthy food labels are seen as perfect tools to optimize consumers' choices over time. Kelly et al. present the critical role that visual design can have in enhancing the effectiveness of nutritional labels. Food products consumers' perceptions can be influenced in a clear manner by the effort to offer easy to understand visual elements, adapted to the communication context of specific food products. Huang et al. propose exploratory research that shed light on consumer psychology—specifically, how nutrition claims can affect Chinese consumers' purchasing decisions. The consumers' need for clear nutritional information is in line with global trends that characterize today's consumers. Modern consumers have become more selective because of the greater access to diversified information sources and literacy, and food products consumption is not an exception. Chilón-Troncos et al. complete these findings with their own research that shows the impact of nutritional literacy on food choices. The study highlighted in a clear manner that informed consumers are more likely to make healthier choices. More and more consumers, from different social and cultural backgrounds are becoming interested in knowing how their food products related decisions can affect their general wellbeing and health status. García-Salirrosas et al. bring a fresh approach to our Research Topic, proposing a research that investigates how perceived value influences brand loyalty. Marketing effectiveness can be reached only through a clear strategy capable of aligning food products brands perception and consumer core values. With the help of clear positioning attributes, brands are capable of differentiation and the promotion of a clear value proposition. Finally, Zaharia and Gonţa discuss the significant impact of social media on body image and dietary behaviors. This paper shows how digital marketing and social media trends can profoundly affect consumer health perceptions and behaviors. Their research brings into our attention the complexity of psychological factors that are determining certain food consumption behavior and interaction with food products, showing the link with important psychological constructs like self-image, body image and social pressure.

Collectively, these articles provide a comprehensive view of how branding, labeling, and marketing influence consumer dietary choices and behaviors, which was the aim of this Research Topic. Further, they explore the way in which perceptions of the value of healthy foods, nutrition labels and health claims influence purchasing decisions and brand loyalty. Factors such as the psychological effects of promoting healthy foods on social media, the importance of label design and visual product promotion as well as the nutritional literacy and the impact of visual messages on consumers are examined. The role of nutritional literacy is highlighted in emerging markets and rural contexts, the role of label design and visual promotion in facilitating or inhibiting the adoption of healthy eating habits being highlighted also. Gathering information and evidence from various research and scientific backgrounds, readers can reach the conclusion that integrating strategic marketing with nutritional literacy and ethical considerations to promote healthier consumer choices and enhance public health outcomes has the outmost importance.
